# Patient-tailored silicone plug for HeartMate 3™ left ventricular assist device explantation

**DOI:** 10.1007/s10047-023-01397-w

**Published:** 2023-04-26

**Authors:** Mohamed Elbayomi, Rene Tandler, Nina Ebel, Dirk W. Schubert, Siegfried Werner, Markus Kondruweit, Micheal Weyand, Christian Heim

**Affiliations:** 1 Department of Cardiac Surgery, Friedrich-Alexander-University, Krankenhausstr. 12, 91054 Erlangen, Germany; 2 Institute for Polymer Materials, Friedrich-Alexander-University, Erlangen, Germany

**Keywords:** Ventricular assist device explantation, HeartMate 3™, Case report

## Abstract

Patient-tailored silicone plug for HeartMate 3™ left ventricular assist device explantation in two successive males proceeded successfully. Given medical therapeutic advancements, FDA-approved plug systems designed by LVAD manufacturers themselves will be necessary for the near future to provide a safe and simple device explantation alternative that fulfills all regulatory standards.

**Supplementary Information:**

The online version contains supplementary material available at 10.1007/s10047-023-01397-w.

## Introduction

**Fig. 1:**
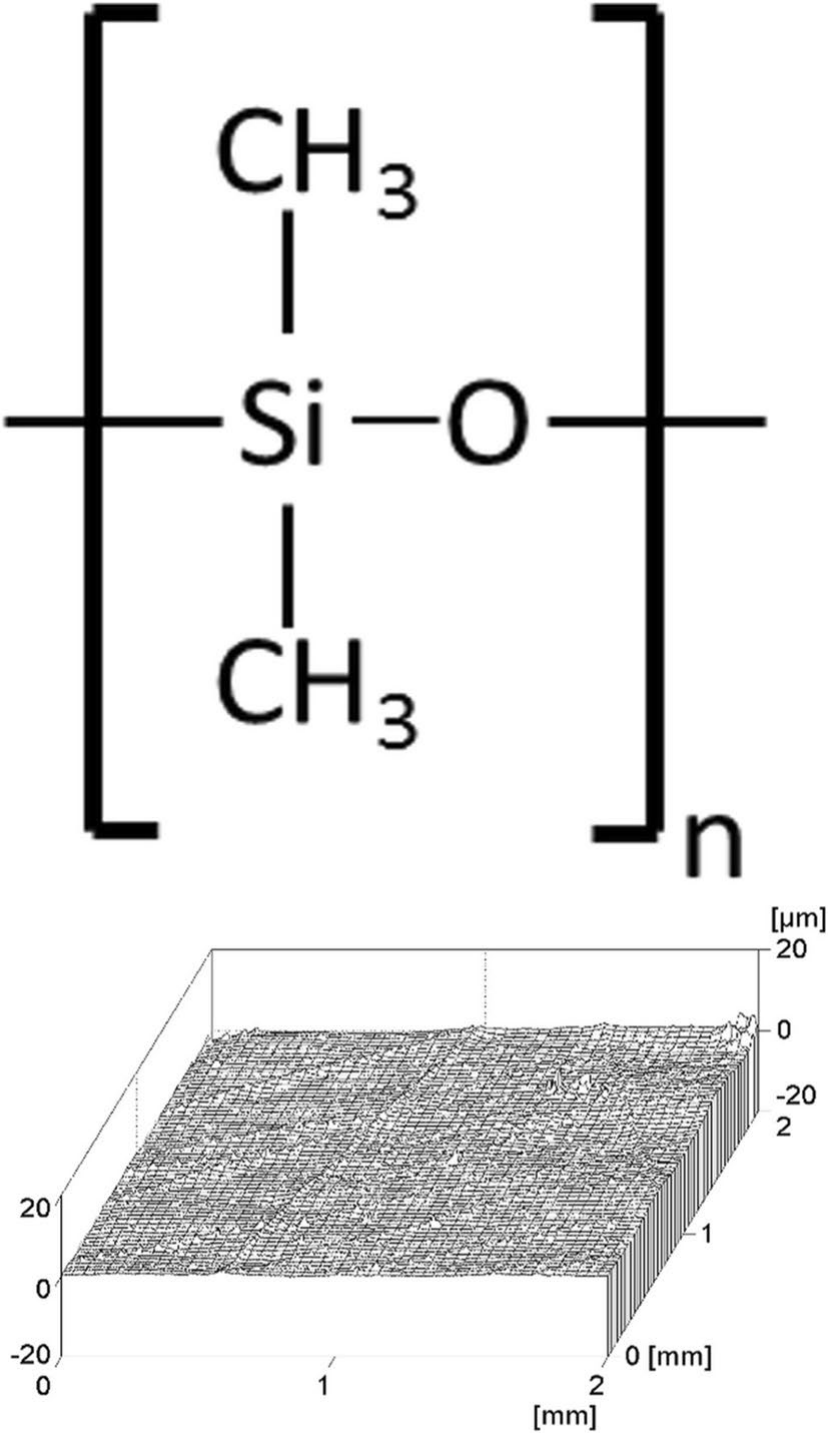
Chemical formula of polydimethylsiloxane (PDMS) and a diagram demonstrating the surface roughness of the silicon plug after applying a laser profilometry with a high-quality average surface roughness (Ra) of 0.13 µm (μm)

The ultimate objective of end-stage heart failure therapy is to restore cardiac function. A recovery after mechanical circulatory support allows explantation of the left ventricular assist device (LVAD). So far, no FDA-approved or CE-certified plug is available for the explantation of the newest generation HeartMate 3™. We present an individualized explantation strategy with an in-house developed patient-tailored silicone plug for the HeartMate 3™ LVAD avoiding (re-) sternotomy in two successive patients. A preoperative CT scan was used to define the precise dimensions of the silicone plug fitting with a HeartMate 3™ apical ring. The cylindrical plug was made from medical grade liquid silicone elastomer MED-4820 (NuSil Silicone Technology LLC, Carpinteria, USA) using a high-quality surface casting mold which is subsequently sterilized in a standard clinical process (Fig. 1). Its geometry implements one rounded end and a cylindrical head with cross-shaped suture guides on the other end.

## Case presentation

Two males in their fifth decade of life with “Stage D” congestive heart failure status and HeartMate 3™ assist device implantation several years prior as a bridge to recovery experienced an improvement of the myocardial function in the follow-up visits, most probably due to lifestyle modification by eliminating the risk factors and strict adherence to guideline-oriented medical treatment. The entire workup of both patients met the mechanical assist device explanation criteria suggested by Berlin Heart Center [1].

Both patients were willing to have a minimally invasive explanation approach through lateral thoracotomy using the recovery plug.

The patients were then prepped and draped in a usual sterile fashion, and a longitudinal left anterior lateral thoracotomy was performed. After careful dissection, the pump and the outflow cannula were identified. Using electrocautery, the pump was mobilized from the surrounding dense adhesion.

After the insertion of an epi-myocardial pacemaker wire to achieve rapid pacing, the screw of the pump was made loose. The lockdown mechanism was opened, the pump was stopped, and the outflow graft was clamped.

**Fig. 2:**
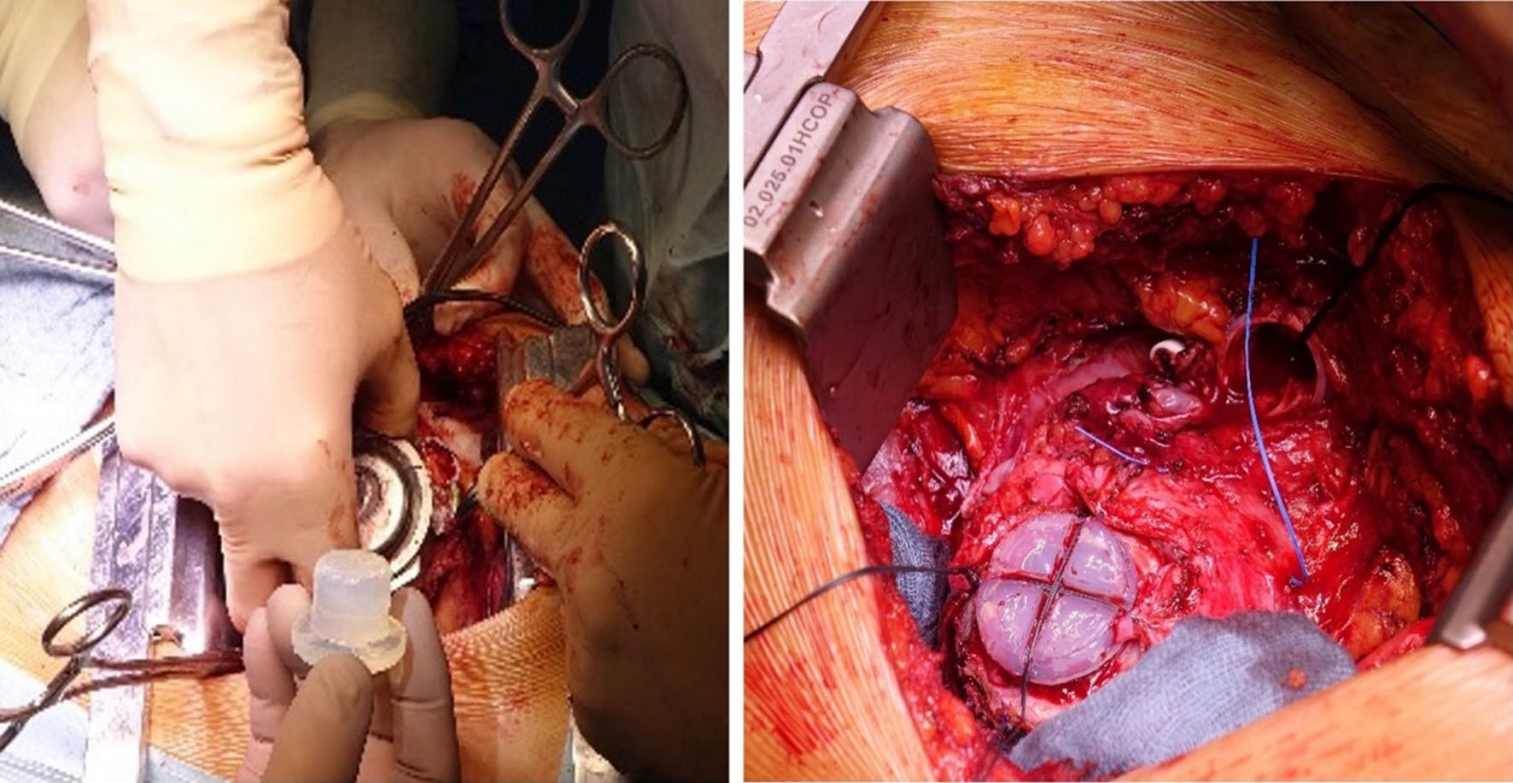
The silicon plug is in the hand of the implanting surgeon, and an intraoperative picture with the silicon plug in situ

After rapid pacing, the pump was removed, and the recovery Plug was swiftly inserted into the sewing ring and then secured (Fig. 2). Afterward, the rapid pacing was terminated, and the drive line was cut with a wire cutter. The pump-related part of the outflow tract and the driveline were removed from the operative field. Using pledgeted 4–0 prolene, the recovery plug was secured and fixed within the myocardium. Using a 3–0 proline, the distal portion of the outflow graft was sewn; then, the clamp was removed. The driveline could be entirely removed from the tunnel. The exit site was packed with iodoform gauze. No apical bleeding occurred after the procedure.

Both patients tolerated all the mentioned procedures well and were monitored in an intensive care unit. After intravenous catecholamine weaning, they were shifted to the surgical floor for further postoperative care, which was uneventful. There was no hospitalization due to heart failure during the follow-up period of four years.

## Discussion

Explantation of LVAD devices is becoming increasingly prevalent as the number of patients receiving LVAD implantation increases and medical treatment advances [2, 3]. Restoring cardiac function is the ultimate objective of congestive heart failure treatment. Up to 10% of individuals with dilative cardiomyopathy regain myocardial function with the insertion of LVAD [4]. Currently, there are no established guidelines or recommendations for assessing recovery during LVAD support. The suggested criteria for weaning and explaining an LVAD are based on echocardiography, electrocardiogram, hemodynamic investigations (e.g., right heart catheterization), and metabolic measurements (spiroergometry) under reduced or no LVAD flow. In both patients, the Berlin Heart Center’s LVAD explanation criteria served as a valid and exhaustive evaluation method [1].

A comprehensive device explant process comprises device deployment, preparation of the apex, removal of the sewing ring, and the employment of Teflon strips to repair the defect in the apex myocardium; this robust approach potentially results in the loss of viable myocardium in a high-risk population. Furthermore, the operating time would be significantly shortened using the recovery plug since there would be no need for ring explantation or oversewing of the left ventricular apex. The omission of cardiopulmonary bypass is also feasible in this presented approach, which might reduce the potentiality of complications. Data from EUROMACS suggests that survival after explantation is excellent without needing a heart transplant or LVAD re-implantation [5]. New FDA-approved plug systems designed by LVAD manufacturers are required in the near future to offer a safe and straightforward device explantation option that meets all regulatory requirements.

### Supplementary Information

Below is the link to the electronic supplementary material.

Supplementary file1 (MP4 28600 KB)

## Data Availability

The raw data supporting the conclusions of this article will be made available by the authors without undue reservation to any qualified researcher.
