# Correction: Patient-tailored silicone plug for HeartMate 3™ left ventricular assist device explantation

**DOI:** 10.1007/s10047-023-01419-7

**Published:** 2023-11-11

**Authors:** Mohamed Elbayomi, Rene Tandler, Nina Ebel, Dirk W. Schubert, Siegfried Werner, Markus Kondruweit, Micheal Weyand, Christian Heim

**Affiliations:** 1 Department of Cardiac Surgery, Friedrich-Alexander-University, Krankenhausstr. 12, 91054 Erlangen, Germany; 2 Institute for Polymer Materials, Friedrich-Alexander-University, Erlangen, Germany

**Correction: Journal of Artificial Organs** 10.1007/s10047-023-01397-w

In this article the author’s name Dirk W. Schubert was incorrectly written as Shubert Dirk.

Also, the given and family names of the authors were incorrectly structured. The correct name should read as “Mohamed Elbayomi, Rene Tandler, Nina Ebel, Dirk W. Schubert, Siegfried Werner, Markus Kondruweit, Micheal Weyand, Christian Heim".

The original article has been corrected.

